# Utility of Biology-Guided Radiotherapy to *De Novo* Metastases Diagnosed During Staging of High-Risk Biopsy-Proven Prostate Cancer

**DOI:** 10.3389/fonc.2022.854589

**Published:** 2022-04-12

**Authors:** Mathieu Gaudreault, David Chang, Nicholas Hardcastle, Price Jackson, Tomas Kron, Gerard G. Hanna, Michael S. Hofman, Shankar Siva

**Affiliations:** ^1^ Department of Physical Sciences, Peter MacCallum Cancer Centre, Melbourne, VIC, Australia; ^2^ Sir Peter MacCallum Department of Oncology, the University of Melbourne, Melbourne, VIC, Australia; ^3^ Department of Radiation Oncology, Peter MacCallum Cancer Centre, Melbourne, VIC, Australia; ^4^ Centre for Medical Radiation Physics, University of Wollongong, Wollongong, NSW, Australia; ^5^ Molecular Imaging and Therapeutic Nuclear Medicine, Prostate Cancer Theranostics and Imaging Centre of Excellence (ProsTIC), Peter MacCallum Cancer Centre, Melbourne, VIC, Australia

**Keywords:** BgRT, PSMA, oligometastasis, prostate, BTZ

## Abstract

**Background:**

Biology-guided radiotherapy (BgRT) uses real-time functional imaging to guide radiation therapy treatment. Positron emission tomography (PET) tracers targeting prostate-specific membrane antigen (PSMA) are superior for prostate cancer detection than conventional imaging. This study aims at describing nodal and distant metastasis distribution from prostate cancer and at determining the proportion of metastatic lesions suitable for BgRT.

**Methods:**

A single-institution patient subset from the ProPSMA trial (ID ACTRN12617000005358) was analysed. Gross tumour volumes (GTV) were delineated on the CT component of a PSMA PET/CT scan. To determine the suitability of BgRT tracking zones, the normalized SUV (nSUV) was calculated as the ratio of SUVmax inside the GTV to the SUVmean of adjacent three-dimensional shells of thickness 5 mm/10 mm/20 mm as a measure of signal to background contrast. Targets were suitable for BgRT if (1) nSUV was larger than an nSUV threshold and (2) non-tumour tissue inside adjacent shell was free of PET-avid uptake.

**Results:**

Of this cohort of 84 patients, 24 had at least one pelvic node or metastatic site disease, 1 to 13 lesions per patient, with a total of 98 lesions (60 pelvic nodes/38 extra-pelvic nodal diseases and haematogenous metastases). Target volumes ranged from 0.08 to 9.6 cm^3^ while SUVmax ranged from 2.1 to 55.0. nSUV ranged from 1.9 to 15.7/2.4 to 25.7/2.5 to 34.5 for the 5 mm/10 mm/20 mm shell expansion. Furthermore, 74%/68%/34% of the lesions had nSUV ≥ 3 and were free of PSMA PET uptake inside the GTV outer shell margin expansion of 5 mm/10 mm/20 mm. Adjacent avid organs were another lesion, bladder, bowel, ureter, prostate, and liver.

**Conclusions:**

The majority of PSMA PET/CT-defined radiotherapy targets would be suitable for BgRT by using a 10-mm tracking zone in prostate cancer. A subset of lesions had adjacent non-tumour uptake, mainly due to the proximity of ureter or bladder, and may require exclusion from emission tracking during BgRT.

## Introduction

Biology-guided radiotherapy (BgRT) is a novel therapeutic modality that intends to guide radiation therapy using functional imaging such as positron emission tomography (PET) (1–3). A linear accelerator incorporating dual 90° PET detectors (PET-linac) has been developed to perform real-time PET image guidance and spatial tracking (RefleXion Medical, Hayward, USA) (4, 5). The PET-linac is equipped with a 6-MV flattening filter-free (FFF) photon beam with a nominal dose rate of 8.5 Gy/min. Dose is delivered by a rotating ring-shape gantry (60 RPM) with an 85-cm bore diameter. The linac includes a multi-leaf collimator (MLC) of 64 leaves, a kVCT imaging system able to acquire 3D CT fan-beam images, a mega-voltage detector array opposite to the linac head, and a 6-degrees-of-freedom couch. The PET-linac may be used to deliver intensity-modulated radiotherapy (IMRT), stereotactic surgery (SRS), and stereotactic ablative radiation therapy (SABR). During BgRT treatment, detection of annihilation photons originating from a volume called the biological tracking zone (BTZ) triggers the delivery of beamlets of radiation to the lesion with sub-second latency. The BTZ ensures that detection of non-target positron emission is minimized. Only the PET signal coming from the BTZ triggers delivery during BgRT.

Prostate-specific membrane antigen (PSMA)-targeted PET tracers have recently been developed for imaging of primary and metastatic prostate cancer (6–11). PSMA PET has been associated with superior specificity and sensitivity compared with conventional imaging (12, 13). Due to its highly specific uptake, there is significant interest in PSMA PET for BgRT of prostate metastases based on PSMA PET.

SABR has been successfully used to treat oligometastatic prostate cancer (OMPC) with excellent local control and minimal toxicity (14–19). There is increasing evidence that delivering ablative doses of radiotherapy to oligometastases can lead to improved survival and ongoing studies are currently evaluating the potential benefit of ablative radiotherapy to polymetastatic diseases (5, 20, 21). BgRT may be ideally suited to the complex task of delivering ablative radiotherapy to polymetastases due to its potential to efficiently treat many lesions in a single session, as well as real-time tracking of tumour motion, which could lead to better sparing of normal tissue (22, 23).

The feasibility of BgRT for OMPC was addressed through a recent planning study (24). It was found that target coverage and conformity were similar between BgRT plans and clinical SABR plans and that BgRT could have efficiency gains because of unified motion management for all lesions.

The goal of this study is twofold. First, we aim to describe disease distribution in synchronous OMPC and synchronous polymetastatic prostate cancer in terms of number of nodal and distant metastases per patient and anatomical location. Second, we aim to characterize the standardized uptake value (SUV) of lesions and their surroundings in order to determine the proportion of lesions that may be suitable for BgRT treatment.

## Materials and Methods

This is a retrospective analysis of PSMA PET/CTs acquired at our institution of patients enrolled in the ProPSMA prospective randomized trial (ID ANZCTR12617000005358) (25, 26). In this trial, patients with high-risk prostate cancer underwent Gallium-68 (^68^Ga) PSMA-11 PET/CT at the time of diagnosis. PET/CT scans were performed with the Discovery PET/CT 690 or 710 (General Electric Medical System, Milwaukee, USA). The PET/CT resolution was 2.9 mm × 2.9 mm × 3.27 mm/1.1 mm × 1.1 mm × 3.27 mm. Lesion identification, node/metastasis classification, and disease staging were assessed by nuclear medicine physicians at the time of diagnosis. Since the ProPSMA clinical trial was a staging study, no radiotherapy planning CTs were acquired for these patients. All patients were androgen deprivation therapy (ADT)-naïve at the time of acquisition.

Guided by the PSMA uptake on PET, nodal or distant metastases were contoured on the CT component of PET/CT by a genitourinary radiation oncologist as gross tumour volume (GTV). This workflow differs from the primary intent of the BgRT workflow where lesions would first be delineated on the planning CT and then the planning CT would be registered to the CT component of a PET/CT scan (4, 5). Segmentation was performed by using the Eclipse treatment planning system (v15.06, Varian Medical Systems, Palo Alto, USA). Lesions were further classified into anatomical categories depending on their location to assess their distribution. Misregistration between the CT component and PET can occur due to a combination of factors including patient movement, respiratory motion, or physiologic movements. If this was the case, the avid region on PET was registered manually to the contour on CT.

The PET signal detected during BgRT treatment must come from the lesion and not from other physiological activity surrounding the lesion to offer reliable spatial tracking. Ideally, a lesion would be isolated from any other physiological activity to be suitable for BgRT. The normalized SUV (nSUV) was calculated to characterize the PET signal. nSUV was defined as the ratio of SUVmax inside the GTV to SUVmean inside an isotropic outer shell margin expansion of the GTV excluding the GTV itself. nSUV is similar to the so-called tumour-to-background ratio (TBR) (27, 28). SUV was normalized by patient body weight to allow for interpatient comparison. ^68^Ga SUV quality control was embedded in the ProPSMA study (29).

The BTZ was the volume resulting from the union of the GTV and the outer shell margin expansion of the GTV. BTZ sizes may vary depending on the lesion size and location. To model the impact of different BTZ sizes, outer shell expansions of 5 mm/10 mm/20 mm were considered. It was assumed that only one lesion could be treated per BTZ.

A lesion may be suitable for BgRT if nSUV is larger than a specific nSUV threshold value; however, the value of the nSUV threshold has not been established for PSMA PET. Potential nSUV threshold values were studied by calculating the cumulative probability distribution function of nSUV to be larger than an nSUV threshold for a lesion. The calculation was repeated for outer shell GTV margin expansions of 5 mm/10 mm/20 mm. Results obtained with nSUV threshold = 3 were explicitly reported in this study for illustration purposes.

A lesion may have nSUV larger than the nSUV threshold but not be suitable for BgRT as high physiological uptake in the BTZ may be averaged out in the determination of SUVmean. Physiological activity inside the BTZ may originate from another avid lesion or from an organ at risk (OAR). To quantify the distance between the neighbouring avid region and the GTV, spherical shells of 3-mm thickness resulting from an outer shell expansion of the GTV were grown at a distance *d* between the outer layer of the shell and the GTV, where *d* ranged from 3 to 50 mm with a 1-mm step size. SUVmax inside these shells as a function of the distance was reported. The proportion of lesions for which SUVmax decreased continuously in a given length interval as a function of the distance from the GTV was determined, and these lesions were designated as isolated lesions. The classification of isolated lesions was repeated by considering uptake increase in 1 to 5 consecutive shells. The optimal number of consecutive shells to consider was determined by manually comparing results with the actual SUV distribution as seen on the PET/CT images. Only SUVmax larger than 1 was considered in the calculation. Outer shell expansions of the GTV and SUV extraction were performed with the MIM software (v6.9.4, MIM Software Inc., Cleveland, OH).

A lesion was considered suitable for BgRT if (1) nSUV was larger than an nSUV threshold and (2) adjacent non-tumour tissue was free of PSMA PET uptake inside the outer shell expansion. Since the value of the nSUV threshold and shell thickness may be variable in the PET-linac system, the nSUV threshold from 2 to 6 and shell expansion with thickness of 5 mm/10 mm/20 mm were reported in this study. 

Differences between distributions were characterized by using the Wilcoxon rank-sum test. The null hypothesis that medians are similar was rejected at the 95% statistical level. Furthermore, statistical correlations were calculated by using the Spearman correlation coefficient (r_s_) and its associated p-value.

## Results

Over the whole patient cohort, PSMA PET/CTs for 84 patients were acquired at our institution. Twenty-four (29%) of these patients had at least one pelvic nodal or one distant metastasis. In these, 98 lesions were segmented, resulting in 60 pelvic nodal diseases (N1) and 38 extra-pelvic nodal diseases and haematogenous metastases (M1).

The most common diagnosis involved at least one node or one metastases (N1M1), which was found in 12 (14%) patients. Twenty (24%) patients had nodal disease (N1M0+N1M1), and 16 (19%) patients had metastatic disease (N0M1+N1M1). This patient distribution was representative of the complete ProPSMA clinical trial cohort [N1M1+N0M1+N1M0: 30.0%, N1M0+N1M1: 25%, N0M1+N1M1: 16%, n = 295 (26)] and similar to another prostate cancer staging study [N1M1+N0M1+N1M0: 32%, N0M1+N1M1: 16%, n = 134 (30)].

**Figure 1:**
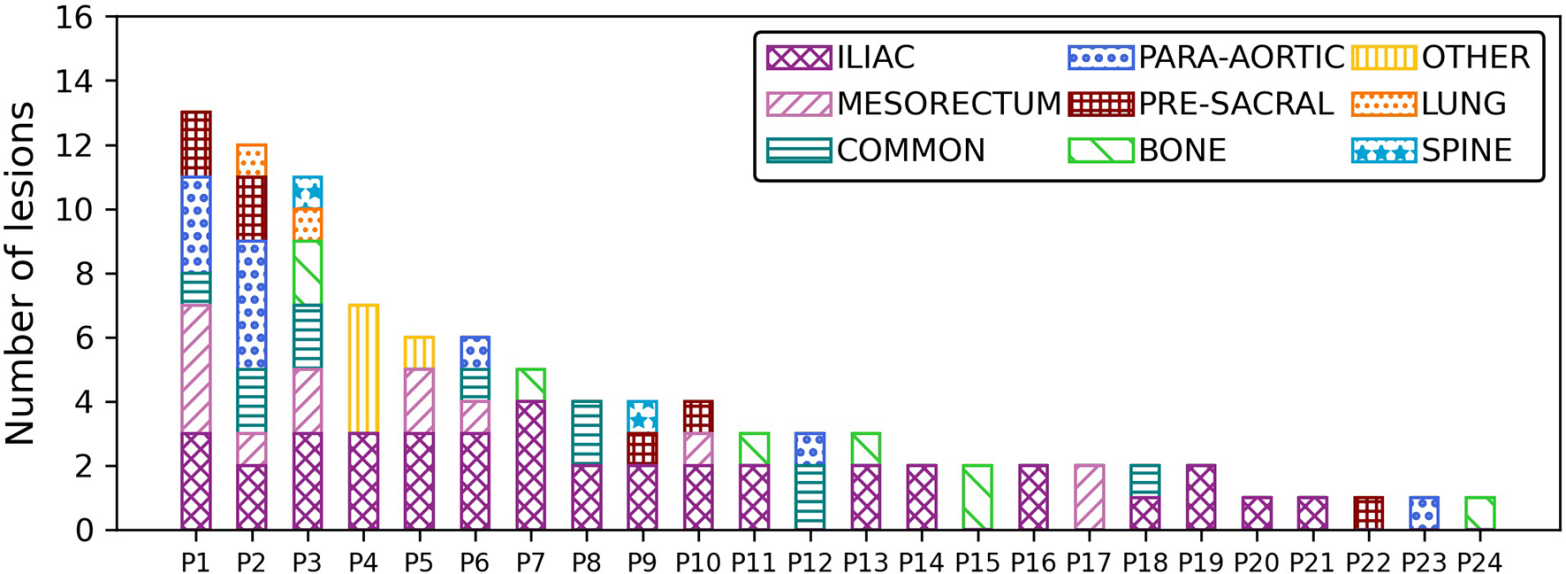
Lesion distribution per anatomical site for the ProPSMA patient cohort.

The distribution of lesions per patient is shown in **Figure 1**. The number of lesions per patient ranged from 1 to 13 lesions, with a median number of 3 lesions per patient. Three patients had more than 10 lesions, and five patients had only 1 lesion. The lesions were further classified into nine categories depending on their anatomical location. The anatomical details of each category are shown in **Table 1**. Lesions were mostly located in the iliac and in the common iliac (52% of all lesions) stations. In addition, a high number of lesions were found in the mesorectum (13%) and in the para-aortic (10%) basin in this population.

**Table 1:** Category used to classify the anatomical location of lesions together with the number of lesions found for each category.

	Category	Anatomical location	Number of lesions
1.	ILIAC	Internal, external, obturator	40
2.	MESORECTUM	Mesorectum	13
3.	COMMON	Common iliac	11
4.	PARA-AORTIC	Para-aortic, interaortocaval	10
5.	PRE-SACRAL	Pre-sacral	7
6.	BONE	Ramus, femur, rib, acetabular	8
7.	OTHER	Inguinal, epigastric	5
8.	LUNG	Intrathoracic, lung	2
9.	SPINE	L3	2

The median three-dimensional registration shift between the CT component and the PET component was less than the PET resolution (median 3D shift = 2.0 mm, interquartile range = 1.3–2.8 mm). Lesion volumes ranged from 0.08 to 9.6 cm^3^ with a median (interquartile range) of 0.76 cm^3^ (0.38–1.4 cm^3^). The difference in volume between the pelvic nodes and the metastases was not statistically significant (p-value = 0.16). SUVmax ranged from 2.1 to 55.0 with median (interquartile range) = 8.6 (4.8–18.0). The SUVmax distribution of pelvic nodes and distant metastases was similar (p-value = 0.60). A positive correlation was observed between SUVmax and the lesion volume (r_s_ = 0.5, p-value < 10^-7^). The correlation was stronger for the pelvic nodes only (r_s_ = 0.6, p-value < 10^-6^) as compared with metastases only (r_s_ = 0.5, p-value < 10^-2^). The calculation was repeated for all anatomical sites. The correlation was statistically significant only in iliac nodes (r_s_ = 0.65, p-value < 10^-5^, n = 40) and bone metastases (r_s_ = 0.83, p-value = 0.01, n = 8).

**Figure 2:**
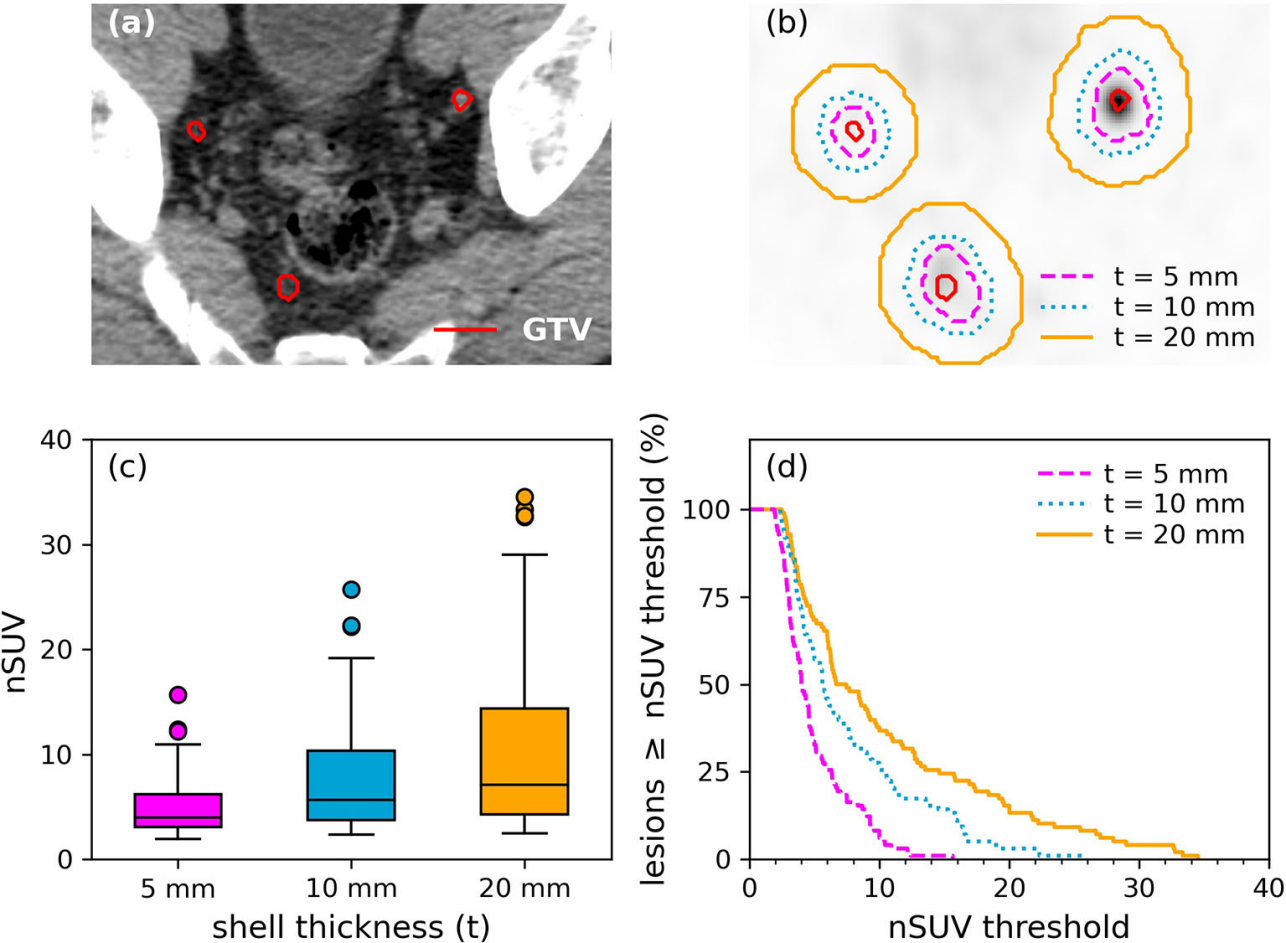
Illustration of lesions for a patient. **(A)** GTVs (red) were segmented on the CT component while **(B)** outer shell expansion was performed on the PET component. Outer shells resulting from a margin expansion of 5 mm/10 mm/20 mm are shown. **(C)** Distribution of nSUV by using an outer shell margin expansion of 5 mm/10 mm/20 mm. **(D)** Cumulative probability distribution function of lesions having nSUV greater or equal to an nSUV threshold as a function of the nSUV threshold. Results inside a shell thickness of 5/10/20 mm are shown.

An illustration of GTV contour on the CT component and its corresponding GTV outer shell margin expansion on the PET component used to determine nSUV is shown in **Figures 2A, B**. The distribution of nSUV calculated for the outer shell margin expansion of 5 mm/10 mm/20 mm is further shown in **Figure 2C**. nSUV increased from a 5-mm margin expansion to a 10-mm margin expansion (p-value < 10^-4^) and from a 10-mm margin expansion to a 20-mm margin expansion (p-value = 0.02) (nSUV = 4.0 (3.1–6.2)/5.7 (3.8–10.3)/7.1 (4.2–14.3), for 5 mm/10 mm/20 mm shell thickness).

The cumulative probability distribution of lesions having nSUV greater or equal to a nSUV threshold as a function of the nSUV threshold is shown in **Figure 2D** for the three outer shell expansions considered. In particular, 76%/88%/93% of the lesions have nSUV ≥ 3 by using a shell expansion of 5 mm/10 mm/20 mm.

**Figure 3:**
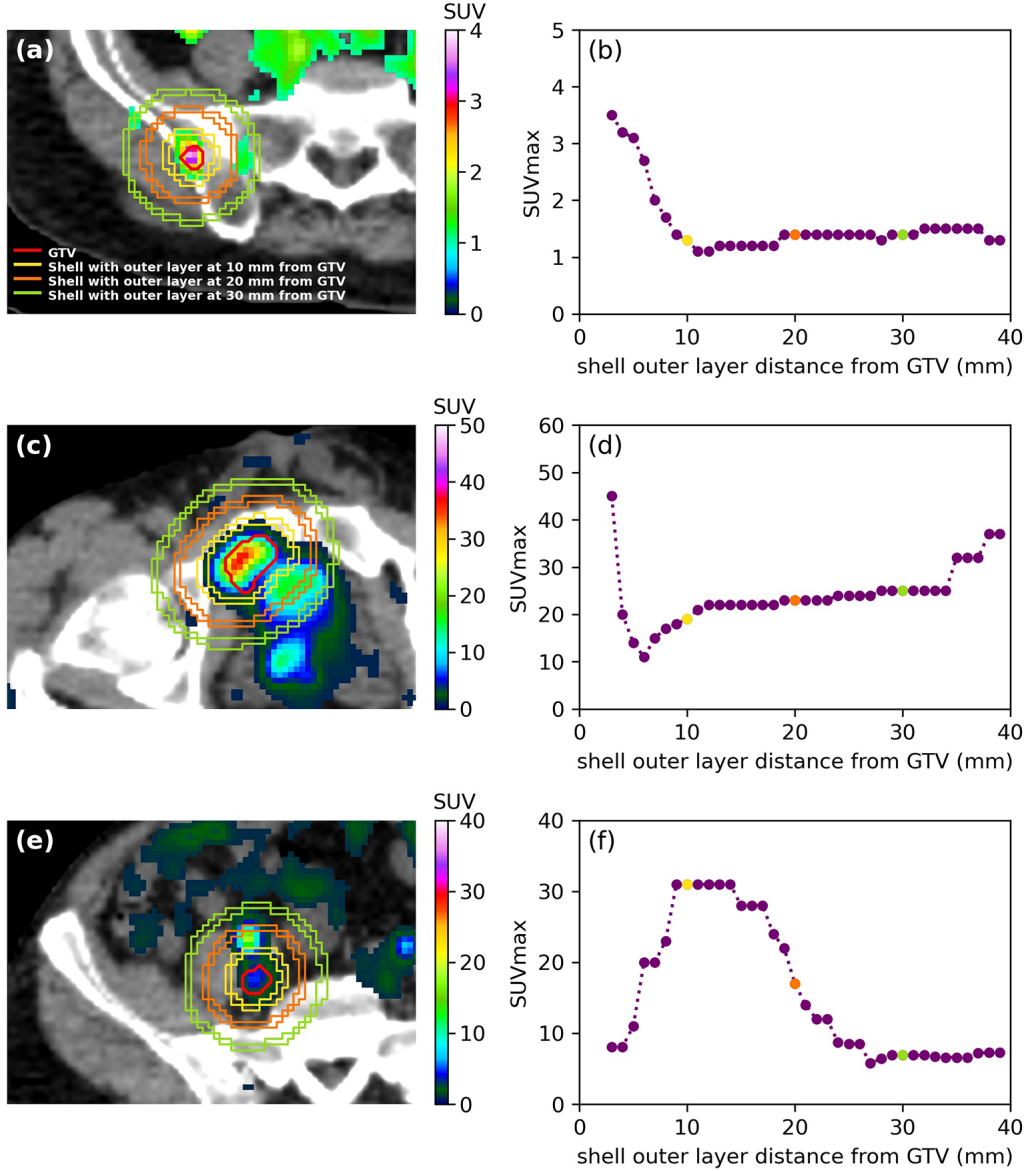
**(A, C, E)** SUV distribution and illustration of the shell method to extract SUVmax for three different patients as well as **(B, D, F)** resulting SUVmax as a function of the distance of the outer later from the GTV (mm). Only SUV *>* 1 is shown for clarity. **(A)** The lesion was isolated from any other functional region, and **(B)** SUVmax decreased or was constant. **(C)** The bladder was located within 10 mm of the lesion, and **(D)** SUVmax increased in the first 5 mm from the lesion. **(E)** The ureter was in the first 5 mm from the lesion, and **(F)** SUVmax increased up to 15 mm away from the lesion.

Examples of spherical shells of fixed 3-mm thickness with the outer layer located at 10 mm/20 mm/30 mm from the GTV are shown in **Figures 3A, C, E** while the extracted SUVmax inside the shell as a function of the distance between the outer layer of the shell and GTV is shown in **Figures 3B, D, F**, respectively. **Figures 3A, B** show an example of an isolated lesion for all distances. However, the bladder was located within the first 10 mm from the GTV in **Figure 3C** and SUVmax increased at distances larger than 6 mm from the GTV, as shown in **Figure 3D**. The ureter was located in the first 5 mm from the GTV in **Figure 3E**, and SUVmax is either increasing or is constant in the first 15 mm from the GTV in **Figure 3F**.

The optimal results to determine if lesions were isolated from any other uptake were obtained when considering a SUVmax increase in two consecutive shells. The ureters were the main avid region near lesions located in the iliac and common iliac (52% of all lesions). The bladder was located at distances larger than 20 mm for lesions in the mesorectum and in other nodes (18% of all lesions). Furthermore, the bowel or another avid lesion was within 15 mm for lesions located in the para-aortic region and in the pre-sacral region (17% of all lesions). Finally, lesions located in the lung, spine, and bone (12% of all lesions) were the most isolated from any other PET signal for all distances.

**Figure 4:**
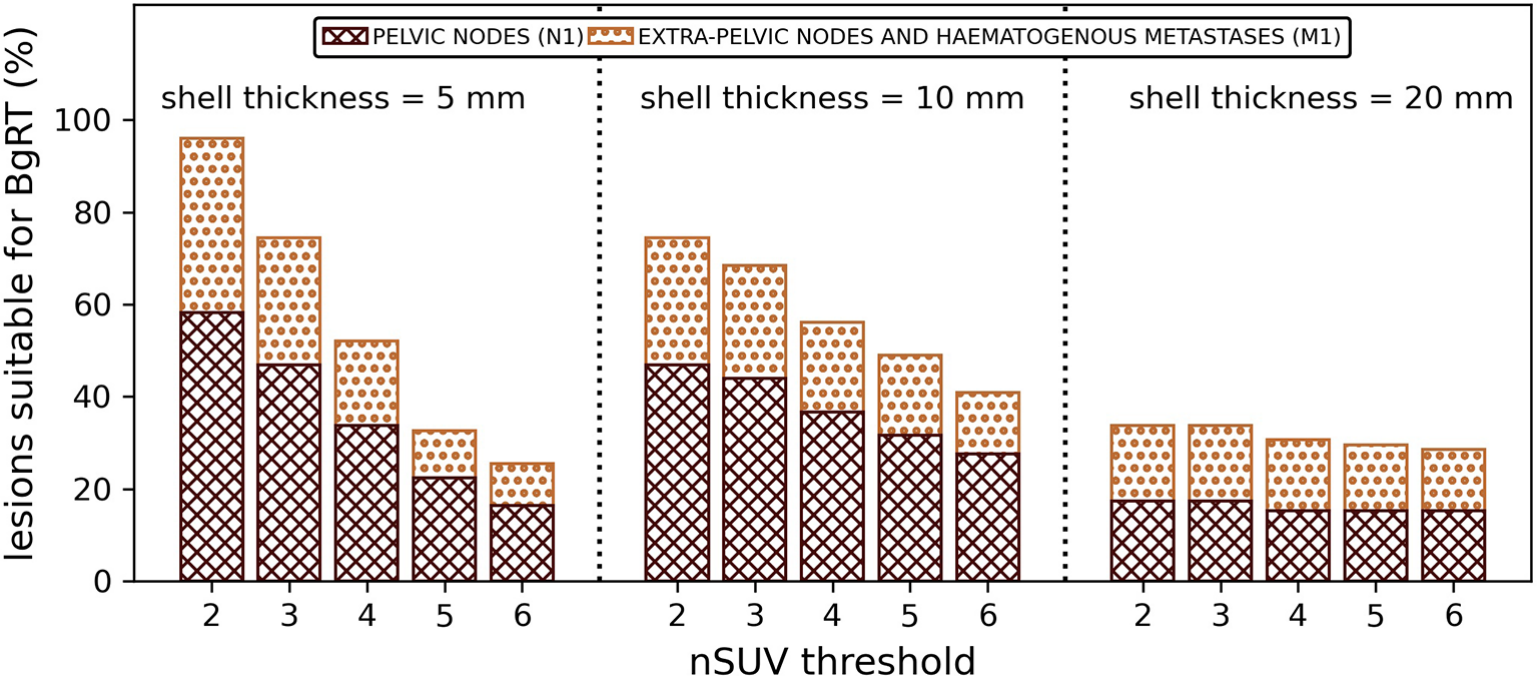
Proportion of lesions (%) suitable for BgRT (nSUV ≥ nSUV threshold and free of PSMA PET uptake inside the GTV outer margin expansion) for several nSUV thresholds. Results per shell thicknesses are shown.

The proportion of lesions suitable for BgRT is shown in **Figure 4**. By using nSUV ≥ 3, 74%/68%/34% of the lesions was suitable for BgRT inside a distance of 5 mm/10 mm/20 mm from the GTV, respectively. The proportion of lesions suitable for BgRT decreased as the threshold was increased; 33%/49%/30% of the lesions was isolated from the adjacent non-tumour uptake and satisfied nSUV ≥ 5 inside the GTV margin expansion of 5 mm/10 mm/20 mm.

## Discussion

BgRT aims at localizing radiotherapy delivery based on biological features and incorporating this information for radiotherapy delivery, simplifying the process of irradiation to multiple sites of disease throughout the body (4, 5). In the context of increasing evidence for ablative radiotherapy for oligometastatic disease, BgRT has a potential role for efficient therapy of metastatic disease in the future (18, 21, 31).

In order to evaluate the feasibility of BgRT in the setting of synchronous oligo- and poly-metastatic metastatic prostate cancer, an anatomical description of the disease for patients enrolled in the ProPSMA clinical trial at our institution was reported. Several BTZ sizes and nSUV thresholds were considered as these parameters may be varied in PET-linac settings.

We have demonstrated that the majority of metastatic targets would have a satisfactory BTZ with a clearly defined tumour. Another lesion, the bladder, or the ureter was commonly found in the surrounding of the lesion. Use of the ^18^F-PSMA-1007 PET tracer which offers a lower urinary clearance and a longer half-life may help to reduce uptake originating from the bladder and the ureter and increase the proportion of lesions suitable for BgRT (32, 33).

Lesions located in the lung, spine, and bone were more isolated from adjacent PET signals when compared with other sites for all distances, suggesting that these locations are optimal candidates for BgRT. However, these locations were less prevalent, representing only 10% of all lesions in this cohort. Lung lesions would benefit from BgRT due to real tracking of lesion motion if margins would be reduced. Spine and bone lesions may benefit from BgRT if the potential of treating multiple lesions in a single session would lead to a significant reduction in the treatment time as compared with an image-guided SABR approach.

This study focused on an early step on the developmental pathway of BgRT treatment, namely, how many lesions would be suitable for BgRT and in what clinical situation. Further steps would be required prior to clinical implementation, which may include the integration of PET in the simulation, treatment planning, and dose calculation processes and determination of the fidelity of the PET distribution immediately before treatment.

There were limitations in this study. First, since the ProPSMA dataset was a staging study, no planning CT was available for this patient cohort. The diagnostic CT component of a PET/CT scan was used to perform the segmentation. This step is not expected to be part of a typical BgRT workflow as a planning CT would be required for delineation. Additional challenges are therefore expected in the BgRT workflow such as accurate registration between the planning CT and the CT component of the PET/CT considering potential changes in anatomy between the two acquisitions or different spatial resolution between the two datasets.

Only one lesion per BTZ was assumed. However, it may be possible to treat multiple lesions inside the BTZ if conditions to suitability are met. Such treatment would increase the number of lesions suitable of BgRT determined in this study since the number of lesions free from adjacent PSMA PET uptake was determined regardless of the source of the PET signal.

It was further assumed that nSUV remained constant from the BgRT planning session to the treatment. nSUV may vary during this period, as observed with androgen deprivation therapy (ADT) (34, 35), and a lesion judged suitable for BgRT during the planning session may not satisfy the suitability conditions at the treatment day. Further studies may assess nSUV robustness through time.

Finally, misregistration between the CT component and the PET component was corrected manually on a per-lesion basis. Misregistration due to patient movement, respiratory motion, and physiological motion is expected to happen during BgRT treatment, and consequently, results presented in this study represent a case scenario where all of the above are accounted for.

## Conclusions

Suitable pelvic nodal and distant metastases for BgRT were identified in this retrospective study for patients with synchronous oligometastatic and synchronous polymetastatic prostate cancer. These lesions are characterized with a high-intensity PET signal inside the GTV and a low-intensity PET signal in their surroundings. Optimal candidates for BgRT were lesions located in the lung, spine, and bone. A subset of lesions had a neighbouring non-tumour uptake due to the proximity of an OAR, which may require exclusion from the biological tracking zone if this option if possible.

## Data Availability Statement

The raw data supporting the conclusions of this article will be made available by the authors, upon reasonable request.

## Author Contributions

All authors contributed to the conception and design of the study. MG and DC analysed and interpreted the patient data. MG performed the statistical analysis. All authors contributed to the manuscript revision and read and approved the submitted version.

## Funding

This research is partially funded by RefleXion Medical. This work is also funded in part by the Peter MacCallum Cancer Centre Foundation. ProPSMA was an Australian and New Zealand Urogenital and Prostate (ANZUP) Cancer Trial Group co-badged study by a clinical trial grant from the Prostate Cancer Foundation of Australia, funded by Movember. Shankar Siva is supported by the Victorian Cancer Council Colebatch Fellowship. MH is supported by a Challenge Award from the Prostate Cancer Foundation (PCF) supporting the Prostate Cancer Theranostics and Imaging Centre of Excellence (ProsTIC); funding was from Canica AS, Oslo, Norway. The funder was not involved in the study design, collection, analysis, interpretation of data, the writing of this article or the decision to submit it for publication.

## Conflict of Interest

The authors declare that the research was conducted in the absence of any commercial or financial relationships that could be construed as a potential conflict of interest.

## Publisher’s Note

All claims expressed in this article are solely those of the authors and do not necessarily represent those of their affiliated organizations, or those of the publisher, the editors and the reviewers. Any product that may be evaluated in this article, or claim that may be made by its manufacturer, is not guaranteed or endorsed by the publisher.
